# MicroRNA-490-3p inhibits colorectal cancer metastasis by targeting TGFβR1

**DOI:** 10.1186/s12885-015-2032-0

**Published:** 2015-12-29

**Authors:** Xuehu Xu, Rong Chen, Zhifa Li, Nanqi Huang, Xiaobing Wu, Shuling Li, Yong Li, Shangbiao Wu

**Affiliations:** Department of Gastrointestinal Surgery, Third Affiliated Hospital of Guangzhou Medical University, Guangzhou, China

**Keywords:** miR-490-3p, Colorectal cancer, Metastasis, TGF-β signaling

## Abstract

**Background:**

Colorectal cancer (CRC) is one of the most common malignances worldwide. Metastasis is responsible for the rapid recurrence and poor prognosis of CRC. However, the underlying molecular mechanism of CRC metastasis remains largely unclear. In this study we purposed to investigate the expression and biological functions of miR-490-3p in CRC metastasis, as well as to identify its downstream target genes and influenced pathway.

**Methods:**

The expression level of miR-490-3p in CRC cell lines, CRC adjacent normal tissues, non-metastasis and metastasis tissues were assessed by quantitative real-time PCR. Patient survivals were follow-up up to 7 years. Gain-of-function and loss-of-function study on cell migration and invasion abilities were carried out by transfection of miR-490-3p mimics or inhibitors respectively. The molecular targets of miR-490-3p were computationally identified and experimentally verified by dual-luciferase reporter assay and western blot. Functional rescue was also conducted to confirm miR-490-3p inhibits CRC metastasis by targeting TGF-β signaling pathway.

**Results:**

miR-490-3p expression was persistently downregulated during CRC malignant progression, as well as in CRC cell lines. Artificially overexpression miR-490-3p in CRC cell lines inhibited cell migration and invasion abilities while knockdown miR-490-3p expression caused the reverse effects. TGFβR1 and MMP2/9 were the downstream targets of miR-490-3p in CRC. Inhibition of TGFβR1 could partially recover the tumor suppression effect of miR-490-3p.

**Conclusion:**

miR-490-3p is downregulated during CRC malignant progression. miR-490-3p represses CRC cell migration and invasion abilities, partially by targeting to the TGF-β signaling pathway. Taken together, miR-490-3p is acting as a tumor suppressor in CRC.

## Background

Colorectal cancer (CRC) is one of the most common gastrointestinal malignance and the third leading cause of cancer-related mortality among males and females worldwide [1]. 90 % of early-stage CRC could be cured by clinical surgery. However, the majority of patients are often diagnosed at an advanced stage thus with poor prognosis [2]. Novel therapeutic targets and diagnostic biomarkers for CRC malignant progression are urgently demanded.

The dysregulation of many oncogenes and tumor suppressor genes has been involved in the tumorigenesis and progression of CRC [3]. In the past decades, a number of microRNAs (miRNAs) serving as oncogenes or tumor suppressors have been demonstrated to be pivotal regulators during tumorigenesis and progression [4]. MiRNAs are a family of small non-coding single strand RNAs ranging from 18 to 25 nt, suppressing gene expression at post-transcriptional level by partial complementary binding to target mRNAs thus resulting in mRNA degradation and/or translational repression. A broad range of biological functions, such as cell proliferation, apoptosis, migration and immune response, were under the precise regulation of miRNAs [5, 6]. Hence, the dysregulation of several miRNAs is very critical for CRC [7]. Among these miRNAs, miR-490-3p has been previously reported to be significantly lower in higher grade ovarian carcinoma. Overexpression of miR-490-3p promoted cell cycle arrest and apoptosis, reduced cell migration and invasion, perhaps by targeting CDK1, Bcl-xL, MMP2/9, CCND1 and SMARCD1 [8]. Similar downregulation of miR-490-3p and its growth- and metastasis-suppressive effects on gastric [9] and lung cancer cells [10] also has been revealed. Even its sibling miR-490-5p, which is originated from one precursor, acts as a tumor suppressor in bladder cancer [11]. However, opposite expression tendency and effects of miR-490-3p was observed in hepatocellular carcinoma (HCC). Elevated expression of miR-490-3p in HCC lead to increased cell proliferation, migration and invasion abilities and contributed to epithelial-mesenchymal transition (EMT) [12]. Confusing dual-faced biological functions of miR-490-3p prompt us to explore its roles in CRC.

The goal of this study was to investigate the expression and biological functions of miR-490-3p in CRC, and to unveil the underlying molecular mechanism of CRC metastasis. We found that the expression of miR-490-3p was significantly decreased in metastasis CRC compared with non-metastasis samples, as well as in CRC cell lines. Overexpression of miR-490-3p in CRC cell line LS174T and HCT116 enhanced cell migration and invasion abilities. We further identified TGFβR1 as a direct target of miR-490-3p, which was confirmed by dual-luciferase reporter assay and western blot. MMP2 and MMP9 were also the downstream targets of miR-490-3p. In general, our study provided evidences to prove that miR-490-3p acts as a tumor suppressor in CRC malignant progression through TGF-β signaling pathway.

## Methods

### Data source

Global miRNA expression profiles of 54 cancerous and 20 non-cancerous colonic tissues were obtained from NCBI Gene Expression Omnibus [GEO: GSE30454] [13]. Candidate differentially expressed miRNAs were identified from the dataset by Student’s t-test analysis as described below. Predicted target genes with binding sites of miR-490-3p were obtained from the TargetScan 6.0 database [14]. TGFβR1 was included in the candidate gene list, containing a conserved miR-490-3p binding site. All the above databases were public and free for use.

### Clinical specimens and cell culture

8 pairs of frozen CRC specimens and matched adjacent non-tumor tissues were histopathologically diagnosed and recruited in the past decade. In addition, another batch of 15 non-metastasis and 15 metastasis CRC tissue samples were also recruited during the time. The tissues were collected at the time of surgery and stored in RNAlater at −80 °C until further use. For the use of the clinical materials for research purposes, informed consent was obtained from each patient, and this study was approved by the ethics committee of the Third Affiliated Hospital of Guangzhou Medical University.

The human CRC cell lines, including HT29, SW620, SW480, HCT-15, LS174T, HCT116, LoVo and DLD-1, were grown in Dulbecco Minimum Essential Medium (DMEM) (Invitrogen, Carlsbad, CA) supplemented with 10 % fetal bovine serum (FBS) (HyClone, Logan, UT) and 1 % penicillin/streptomycin. Cells were maintained in a humidified atmosphere at 37 °C with 5 % CO_2_.

### RNA extraction and quantitative real-time PCR

To measure the expression level of miR-490-3p, total miRNA from cultured cells or surgical specimens was extracted using the mirVana miRNA Isolation Kit (Ambion, USA) according to the manufacturer’s instructions. cDNA was synthesized from 5 ng of total miRNA using the TaqMan miRNA reverse transcription kit (ABI, USA), and the expression levels of miR-490-3p were quantified using miRNA-specific TaqMan MiRNA Assay Kit (ABI, USA) in an ABI 7500 real-time PCR machine. U6 small nuclear RNA was used as an endogenous control.

To measure the mRNA levels of MMP2 and MMP9, total RNA extracted using Trizol reagent (Invitrogen, USA) was reversely transcribed using the ImProm-II Reverse Transcription System (Promega, USA). Quantitative real-time PCR was performed by using SYBR Green PCR master mix (Roche, USA) on an ABI 7500 real-time PCR machine. The primers selected were as the following: MMP2 forward: 5′- AGGCCAAGTGGTCCGTGTGA-3′, reverse: 5′-TAGGTGGTGGAGCACCA GAG-3′; MMP9 forward: 5′- ATCCGGCACCTCTATGGTCCTC-3′, reverse: 5′- GCACAGTAGTGGCCGTAGAAGG-3′; expression data were normalized to the geometric mean of housekeeping gene β-actin to control the variability in expression levels (forward: 5′-TGGCACCCAGCACAATGAA-3′; reverse primer, 3′-CTAAGTCATAGTCCGCCTAGAAGCA -5′).

### Plasmids construction

The full-length sequence of TGFβR1 3′ untranslated region (3′UTR) is 4888 bp long and contains one conserved miR-490-3p binding sites from 3933 bp to 3939 bp. The region of human TGFβR1 3′UTR, from 3780 to 4176 bp, generated by PCR amplification from genomic DNA, were cloned into the Kpn I/Xho I sites of the pGL3-basic luciferase reporter plasmid (Promega, USA). The primers selected were as the following: TGFβR1-3′UTR forward: 5′- CGGGGTACCGTTGTGCCAACGGAATAGGG -3′; reverse: 5′- CCGCTCGAGCTCCTCTTTACAGGCTTCTCAG -3′. Mutant inserts containing substitutions in the miRNA complementary sites were generated by PCR using the primers: TGFβR1-3′UTR-mut forward:5′- CATACTTTATAGAAATAAAACTGCACGATTGGAGAATGCTCTGACAAATATTAAAC-3′ and reverse,5′- GTTTAATATTTGTCAGAGCATTCTCCAATCGTGCAGTTTTATTTCTATAAAGTATG -3′; PCR products were cloned into the modified pGL3 control vector (Promega, USA) immediately downstream of the stop codon of the luciferase gene. Wild-type and mutant inserts were confirmed by sequencing.

### Cell transfection

The miR-490-3p mimics (miR-490-3p), inhibitors (miR-490-3p-in) and their relative negative controls (NC), accompanied with TGFβR1 siRNA were purchased from RiboBio (RiboBio Co.Ltd, Guangzhou, China). Transfection of microRNA or microRNA inhibitor or their relative controls were performed using the Lipofectamine 2000 reagent (Invitrogen, USA) according to the manufacturer’s instruction. The final concentration of transfection was 20 nM if not specified.

### Wound healing assay

Cell migration ability was measured using the scratch assay. Briefly, cells were seeded on six-well plates with DMEM containing 10 % FBS and grown to monolayer confluence. The cell monolayers were scratched with a sterile pipette tip to create straight wounds. At 0 and 24 h after wounding, respectively, migration images were captured and documented at different time points using an inverted Olympus IX50 microscope with 10× objective lens and the Image-Pro Plus software (Media Cybernetics).

### Transwell matrix penetration assay

For migration and invasion assays, cells (2 × 10^4^) to be tested were plated on the top side of the polycarbonate Transwell filter with Matrigel (BD Biosciences, San Jose, CA) coating in the upper chamber of the BioCoat^TM^ Invasion Chambers (BD, Bedford, MA, USA) and incubated at 37 °C for 24 h, followed by removal of cells inside the upper chamber with cotton swabs. Invaded cells on the membrane bottom-surface were fixed in 1 % paraformaldehyde, stained with 0.2 % (w/v) crystal violet solution for 15 min, and Cells adhering to the undersurface of the filter were counted (Ten random 100× fields per well) using an inverted microscope. Three independent experiments were performed and the data are presented as mean ± standard deviation (SD).

### 3D morphogenesis assay

24-well dishes were coated with Growth Factor Reduced Matrigel (BD Biosciences, California, USA), and covered with growth medium supplemented with 2 % Matrigel. Cells were trypsinized and seeded at a density of 1 × 10^4^. The medium was replaced with 2 % Matrigel every 3 to 4 days and microscopic images (200x magnifications) were captured at 2 day intervals for 2 to 3 weeks.

### Dual-luciferase reporter assay

HEK-293 T cells (4 × 10^4^) were seeded in triplicates in 24-well plates and allowed to settle for 24 h. And then 100 ng of pGL3- TGFβR1-3′UTR(wt/mut), or the control-luciferase plasmid, plus 1 ng of pRL-TK renilla plasmid (Promega, Madison, WI), were transfected into the cells using Lipofectamine 2000 reagent (Invitrogen Co., Carlsbad, CA) according to the manufacturer’s recommendation. The firefly and renilla luciferase signals were measured 48 h after transfection using the Dual Luciferase Reporter Assay Kit (Promega, Madison, WI) according to the manufacturer’s protocol. Three independent experiments were performed and the data are presented as the mean ± SD.

### Western blot

The western blot analysis was performed according to standard methods as previously described [15], using anti-TGFβR1 antibodies (Abcam, Cambridge, MA). The membranes were stripped and reblotted with an anti-β-actin monoclonal antibody (Sigma, Saint Louis, MO) as a loading control. The relative Western blot bands density was measured by computational software ImageJ (which has been widely used to compare the density of bands on an agar gel or Western blot) and normalized with sample T1 [16].

### Statistical analysis

Statistical analysis was performed using SPSS 16.0 software (SPSS Inc., USA), and the data were presented as means ± SD. A two-tailed t-test was used to evaluate the statistical significance of the differences between two groups of data in all pertinent experiments in this study. Survival analysis was carried out with the Kaplan-Meier method. A *P*-value < 0.05 was considered to be statistically significant.

## Results

### Decreasing miR-490-3p correlates with CRC tumorigenesis and progression

**Fig. 1:**
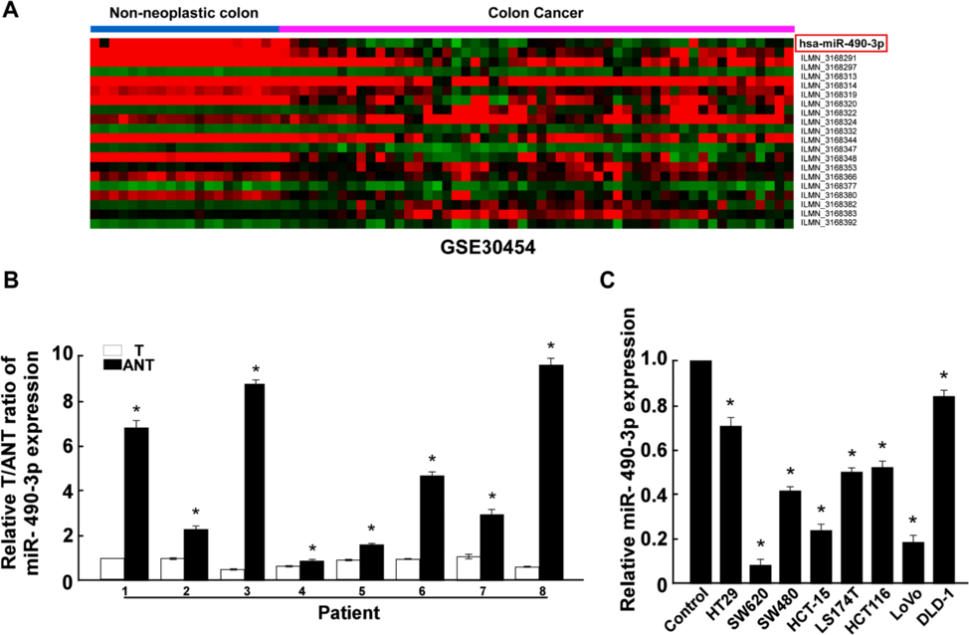
Downregulation of miR-490-3p in human CRC tissues and cell lines. **a** Global miRNA expression profile revealed miR-490-3p was downregulated in CRC tissues versus non-neoplastic colon. Each row of the heatmap indicated one tissue sample and each column indicated one miRNA. Labels in the right represented for miRNA symbol or probeset. Red: high expression; Green: low expression. **b** Quantitative real-time PCR analysis of miR-490-3p expression in 8 pairs of CRC tumor and adjacent non-tumor tissue samples. **c** Quantitative real-time PCR analysis of miR-490-3p expression in 8 CRC cell lines. *: A two-tailed t-test *P*-value <0.05

**Fig. 2:**
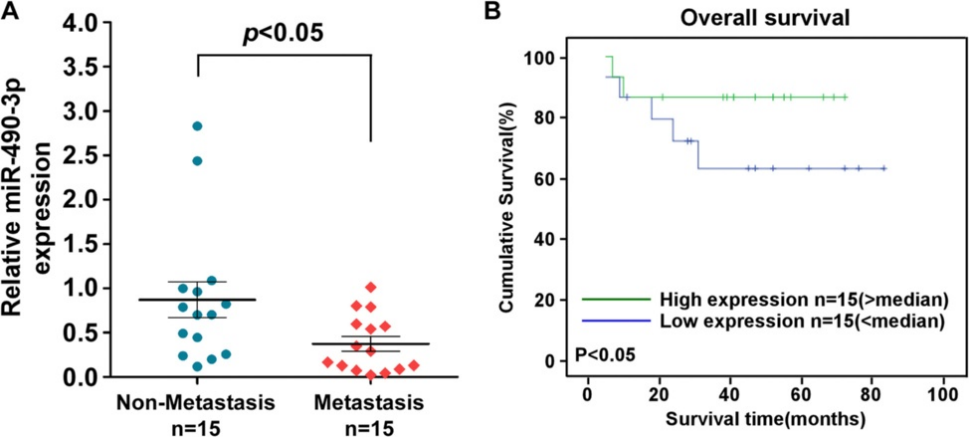
miR-490-3p expression persistently decreased during CRC progression. **a** Quantitative real-time PCR analysis of miR-490-3p expression in 15 metastasis CRC samples in comparison with the expression in 15 matched non-metastasis CRC samples. **b** Overall survival curve indicated that lower expression level of miR-490-3p was associated with poor prognosis of CRC

As described before, dual-faced functional roles of miR-490-3p have been found in various cancers, however, its correlation with CRC remains largely unknown, which prompt us to explore its expression and function in CRC. After data mining of previously reported global miRNA expression profiles of 54 cancerous and 20 non-cancerous colonic tissues [13], we identified a miRNA, miR-490-3p, was downregulated in majority of CRC samples (Fig. 1a). By quantitative real-time PCR determination, we confirmed the decreasing expression of miR-490-3p in 8 pairs of CRC tumor and adjacent non-tumor tissue samples (Fig. 1b), as well as in all the 8 CRC cell lines (HT29, SW620, SW480, HCT-15, LS174T, HCT116, LoVo, DLD-1) analyzed in this study (Fig. 1c, *P* < 0.05). Although wide expression discrepancies had been found among these patient samples and cell lines, individual heterogeneity and the complexity of clinical samples (including different tumor stage, tumor size, patient age and gender, and etc.) still do not mask this decreasing tendency. Moreover, miR-490-3p expression was further downregulated in fifteen metastasis CRC samples, in comparison with the expression level in fifteen matched non-metastasis CRC samples (Fig. 2a, *P* < 0.05). Following-up of these patients indicated lower expression level of miR-490-3p was associated with poor prognosis of survival (Fig. 2b, *P* < 0.05). Taken together, miR-490-3p expression was downregulated during CRC tumorigenesis and malignant progression. miR-490-3p perhaps acts as a tumor suppressor in CRC, which would be determined below.

### miR-490-3p suppresses CRC cell migration and invasion abilities

To investigate the biological functions of miR-490-3p in CRC, LS174T and HCT116 cell lines were used for gain-of-function and loss-of-function studies. The medium-level of miR-490-3p expression in the two cell lines were suitable for either overexpression or knockdown modification of the same genetic background (Fig. 1c). After overexpression of miR-490-3p by transfection of 20 nM synthesized mimics, or knockdown of miR-490-3p by transfection of 20 nM synthesized inhibitors, LS174T and HCT116 cell migration and invasion abilities were assessed in comparison to the cells transfected with 20 nM NC respectively.

**Fig. 3:**
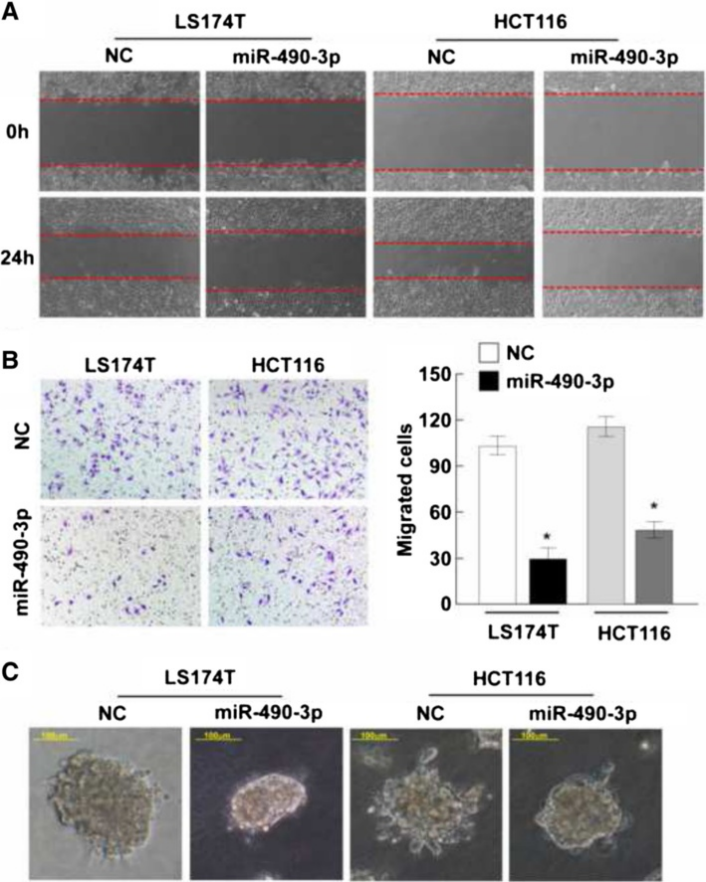
Overexpression of miR-490-3p suppresses CRC cell migration and invasion abilities. **a** Wound healing assay was used to assess the mobile ability of CRC cells (LS174T and HCT116) transfected with miR-490-3p mimics or negative control respectively. miR-490-3p inhibited the wound closure obviously (magnification: 100x). **b** Matrigel transwell assay revealed miR-490-3p significantly reduced the number of invaded CRC cells (*P* < 0.05). **c** Cell 3D morphology showed that LS174T and HCT116 CRC cells with increased expression of miR-490-3p presented less outward projections and spheroid morphology, which was associated with an impaired aggressive ability (magnification: 200x). *: A two-tailed t-test *P*-value <0.05

**Fig. 4:**
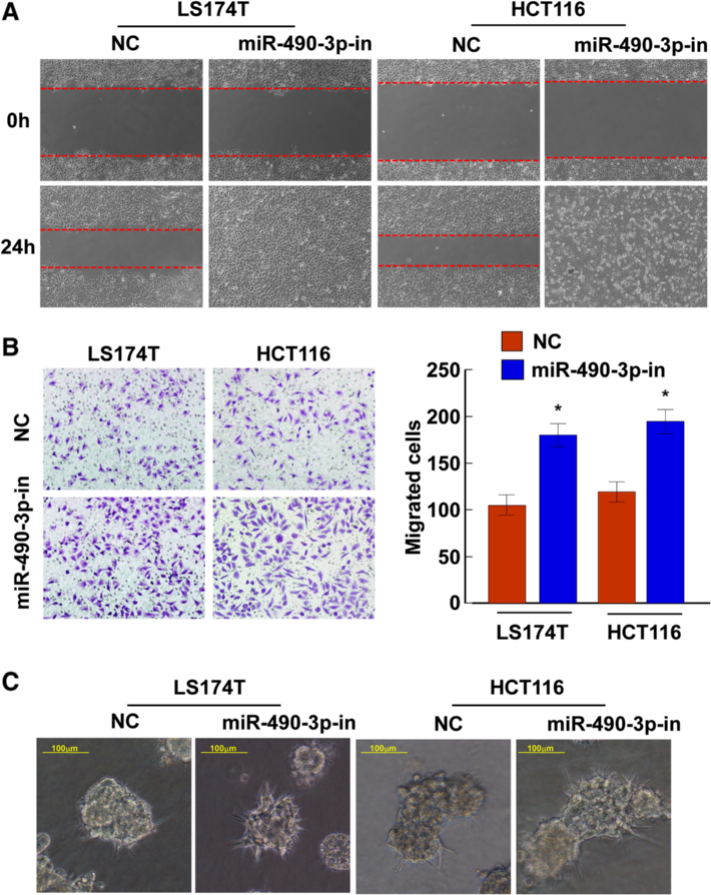
Knockdown of miR-490-3p promotes CRC cell migration and invasion. **a** Wound healing assay was used to assess the mobile ability of CRC cells (LS174T and HCT116) transfected with miR-490-3p inhibitors or negative control respectively. Knockdown of miR-490-3p expression markedly accelerated the wound healing (magnification: 100x). **b** Transwell matrix penetration assay revealed knockdown of miR-490-3p expression significantly increased the number of invaded CRC cells (*P* < 0.05). **c** Cell 3D morphology showed that LS174T and HCT116 CRC cells with decreased expression of miR-490-3p presented more outward projections and irregular shapes, which was associated with an highly aggressive ability (magnification: 200x). *: A two-tailed t-test *P*-value <0.05

By wound healing assay, we found that overexpression of miR-490-3p slowed down the confluence of LS174T and HCT116 cells (Fig. 3a), while knockdown of miR-490-3p promoted the wounds healing reversely (Fig. 4a). Similar effects were observed in Matrigel transwell assay. Overexpression of miR-490-3p dramatically reduced the number of invaded cells on the membrane bottom-surface (Fig. 3b, *P* < 0.05) and knockdown of miR-490-3p increased the number of invaded cells (Fig. 4b, *P* < 0.05). All the above observations suggested miR-490-3p could suppress CRC cell migration and invasion abilities.

In addition, in a 3D morphogenesis assay, LS174T and HCT116 cells with increased expression of miR-490-3p presented less outward projections and spheroid morphology compared to the negative control (Fig. 3c), which was associated with an impaired aggressive ability. Contrary phenotype was observed in CRC cells with decreased expression of miR-490-3p (Fig. 4c).

### miR-490-3p targets TGFβR1 in CRC cells

**Fig. 5:**
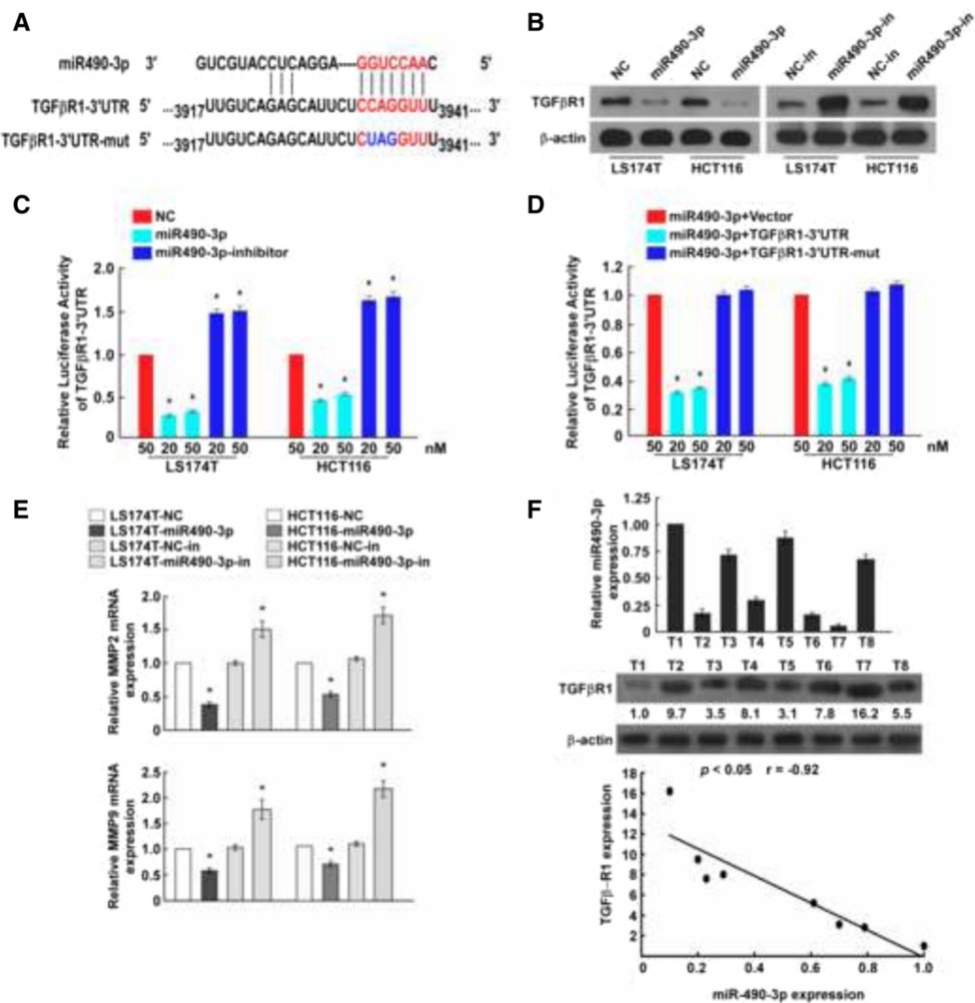
miR-490-3p targets TGFβR1 and MMP2/9 in CRC cells. **a** TGFβR1 was predicted to be a theoretical miR-490-3p target. Complete base pairing in seed region of TGFβR1 3‘UTR was disrupted in constructed mutant plasmid to verify direct interaction. **b** Western blot results showed that overexpression of miR-490-3p in CRC cell lines decreased the protein level of TGFβR1, and knockdown expression of miR-490-3p increased the protein level of TGFβR1 inversely. **c** Dual-luciferase reporter assay indicated miR-490-3p bound to TGFβR1 3‘UTR directly. **d** Disruption of seed region base pairing blocked the interaction between miR-490-3p and TGFβR1. **e** Quantitative real-time PCR analysis suggested MMP2 and MMP9 were the downstream responses of miR-490-3p interference. **f** The relative miR-490-3p expression and TGFβR1 protein level in 8 CRC tissues were quantified by real-time PCR and western blot respectively. Linear regression analysis of them revealed a strong negative correlation. *: A two-tailed t-test *P*-value <0.05

By using a popular miRNA targets prediction algorithm TargetScan, TGFβR1 was predicted to be a theoretical target of miR-490-3p (Fig. 5a), which miRNA recognition element (MRE) in 3′UTR was conserved among mammals. Western blot results showed that overexpression of miR-490-3p in CRC cell lines decreased the protein level of TGFβR1, and knockdown expression of miR-490-3p increased the protein level of TGFβR1 inversely (Fig. 5b). Dual-luciferase reporter assay indicated that miR-490-3p suppressed TGFβR1 expression by direct binding to the MRE (Fig. 5c), while mutants in the seed region disrupted their interaction (Fig. 5d). MMP2 and MMP9 have been previously reported to be direct target genes of miR-490-3p in ovarian cancer [8]. Here we found that MMP2 and MMP9 mRNA expression was downregulated after overexpression of miR-490-3p and upregulated after knockdown of miR-490-3p (Fig. 5e), confirming the interactions between them. Moreover, in eight CRC patient tissue samples, a significant reversed correlation between miR-490-3p expression and TGFβR1 protein level had been identified (Fig. 5f, r = −0.92, *P* < 0.05), which further proved the true inhibitory relationship.

### Inhibition of TGFβR1 partially recovers the tumor suppression function of miR-490-3p

**Fig. 6:**
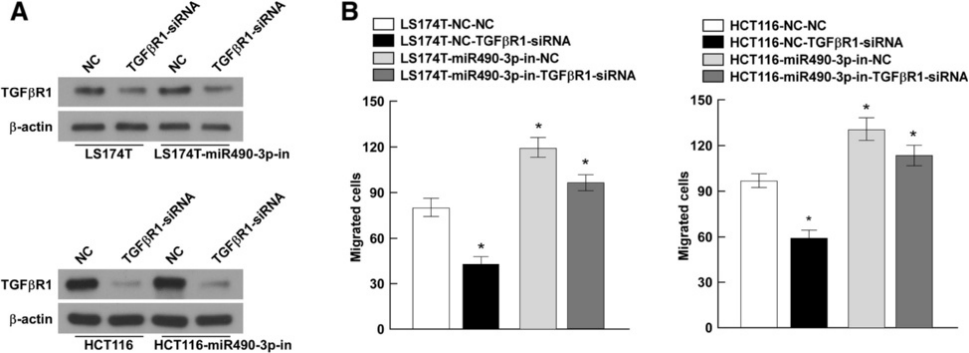
Inhibition of TGFβR1 partially recovers the tumor suppression function of miR-490-3p. **a** CRC cells (LS174T and HCT116) were treated with TGFβR1 siRNA, miR-490-3p inhibitors, or both of them. Western blot presented the TGFβR1 protein level after interference. **b** Cell migration ability was assessed by transwell matrix penetration assay after interference. Co-transfection of TGFβR1 siRNA and miR-490-3p inhibitors partially but not completely reduced the malignant potency caused by miR-490-3p inhibitors. *: A two-tailed t-test *P*-value <0.05

To further investigate the functional consequence of the miR-490-3p/TGFβR1 interactions in CRC, we compared the migration abilities of CRC cells treated with TGFβR1 siRNA, miR-490-3p inhibitors, or both of them. TGFβR1 siRNA markedly reduced the TGFβR1 protein level, while miR-490-3p inhibitors elevated the TGFβR1 protein level. Co-transfection of TGFβR1 siRNA and miR-490-3p inhibitors partially recovered TGFβR1 expression when compared to the cells only transfected with siRNA (Fig. 6a). Meanwhile, the cell migration ability was assessed by transwell matrix penetration assay for the same treatment. Transfection of TGFβR1 siRNA decreased the migrated cells and transfection of miR-490-3p inhibitors increased the migrated cells respectively. Co-transfection of them partially but not completely reduced the malignant potency caused by miR-490-3p inhibitors (Fig. 6b). Taken together, evidences indicated inhibition of TGFβR1 could partially recover the tumor suppression effect of miR-490-3p.

## Discussion

Human miR-490-3p gene is located in chromosome 17 (136903167-136903294 [+], GRCh38), highly conserved among mammals, indicating a fundamental and pivotal function role during evolution [17]. However, researches concerning miR-490-3p are still very limited. Current knowledge emphasizes its relevance to diseases in particular various types of cancer. As described above, miR-490-3p is likely to be a tumor suppressor in ovarian [8], gastric [9] and lung cancer [10] cells but acts as an oncogenic factor in liver cancer on the contrary [12]. Such dual-faced biological functions have been discovered in many miRNAs, such as miR-503-5p [18–20] and etc. Dysregulation of miR-490-3p in CRC has been found in one large scale screening yet [13], therefore, it’s worthy of further investigation to unveil its roles in CRC.

TGFβR1 protein is a membrane serine/threonine protein kinase. It forms a heteromeric complex with type II TGF-β receptors when bound to TGF-β, transducing the TGF-β signal from the cell surface to the cytoplasm. TGF-β signaling inhibits cell cycle G1/S transition in the early stage of tumor onset but promotes cell migration and invasion in advanced stage of tumor [21]. In the present study, we found miR-409-3p was downregulated not only during CRC tumorigenesis but persistently decreased during CRC development. miR-490-3p inhibits cell migration and invasion in part by direct targeting to TGFβR1 and MMP2/9. Impaired expression of miR-490-3p during CRC development led to increasing TGFβR1. And then activated TGFβR1 phosphorylates SMAD2 which dissociates from the receptor and interacts with SMAD4. The SMAD2-SMAD4 complex is subsequently translocated to the nucleus where it modulates the transcription of the TGF-β-regulated genes [22]. Interestingly, TGF-β signaling is also double-edged for cancer development [21]. TGF-β signaling functions as tumor suppressor on the early stage of tumorigenesis by causing cell cycle arrest and inducing apoptosis. But over activated TGF-β signals are often observed in many advanced carcinomas, which have been correlated with increased tumor invasiveness and malignant progression. Regulation of TGFβR1 by miRNAs had been reported in some other diseases such as non-small cell lung cancer [23]. We proved that decreased miR-490-3p in CRC cells result increased protein level of TGFβR1 which enhanced CRC migration and invasion by elevating TGF-β signals. Besides directly promoting cell migration and invasion, TGF-β signaling is also involved in EMT [24, 25], tumor angiogenesis [26], and immunosuppression [27], which are the key procedures of cancer metastasis. We supposed that miR-490-3p might also regulate EMT, angiogenesis and immune response in CRC progression. Limited to our current capacity, this hypothesis would be verified next time.

It’s known that one miRNA could target to hundreds of genes, forming a complex regulating network [14]. Here we identified TGFβR1 as a novel miR-490-3p target. In addition, the inhibitory effects of miR-490-3p on the mRNA level of MMP2/9 had been validated in CRC cells. Degradation of the extracellular matrix is a critical event during the malignant progression of cancer, which requires a number of extracellular enzymes like matrix metalloproteinase (MMP) [28]. MMPs have been recognized as involved in cancer invasion and metastasis, especially MMP2 and MMP9 [29, 30]. Elevated MMP2 and MMP9 expression promotes the degradation of type IV collagen, a major constituent of basement membranes. Moreover, noticing that TGFβR1 siRNA only partially rescued the malignant consequence caused by miR-490-3p downregulation (Fig. 6), there is reason to believe that additional genes beyond TGF-β signaling pathway should be included in the miR-490-3p regulating network.

## Conclusion

In conclusion, our studies demonstrate miR-490-3p is persistently downregulated during CRC malignant progression. miR-490-3p inhibits CRC cell migration and invasion abilities in part by targeting to TGFβR1 and MMP2/9, therefore interfering TGF-β signaling transduction. Taken together, miR-490-3p is acting as a tumor suppressor in CRC.

## Abbreviations

3′UTR: 3′ untranslated region
CRC: colorectal cancer
DMEM: Dulbecco minimum essential medium
EMT: epithelial-mesenchymal transition
FBS: fetal bovine serum
GEO: gene expression omnibus
HCC: hepatocellular carcinoma
miRNA: microRNA
MMP: matrix metalloproteinase
MRE: miRNA recognition element
NC: negative controls
SD: standard deviation
